# Supplementary material for: Chemogenetic inactivation reveals the inhibitory control function of the prefronto-striatal pathway in the macaque brain
Source: Commun Biol. 2021 Sep 16;4:1088. doi: 10.1038/s42003-021-02623-y (PMC8446038; doi:10.1038/s42003-021-02623-y)
Supplement: Supplementary file 2 — Supplementary information [file 42003_2021_2623_MOESM2_ESM.pdf]

Supplementary Information

Chemogenetic inactivation reveals the inhibitory control function of the prefronto-striatal pathway in the macaque brain

Mineki Oguchi, Shingo Tanaka, Xiaochuan Pan, Takefumi Kikusui, Keiko Moriya-Ito, Shigeki Kato, Kazuto Kobayashi, Masamichi Sakagami

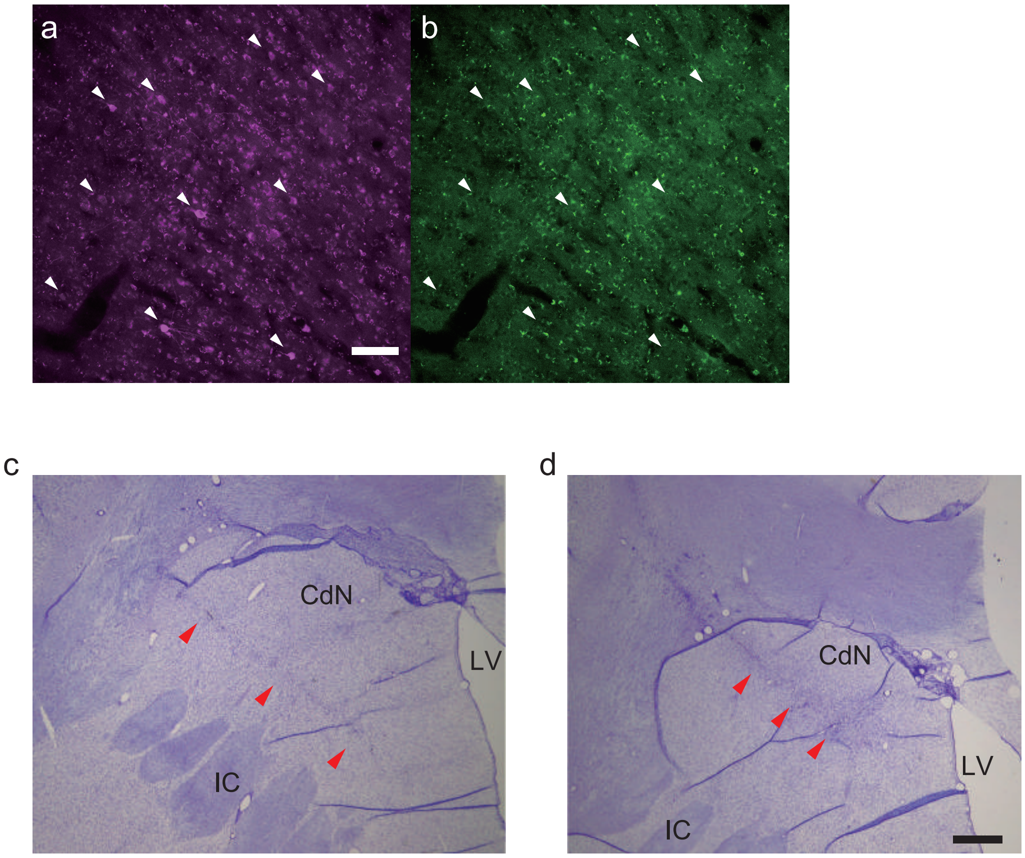

Supplementary figure 1 | mCherry positive cells in the LPFC and needle trace in the CdN of Monkey W

(**a**) mCherry-positive cells in the right LPFC of Monkey W, as observed using a WIG filter cube. White arrows indicate mCherry-positive cells. Scale bar: 100 µm. (**b**) The micrograph of the same area as in (a) observed using a NIBA filter cube. (**c**) (**d**) Nissl-stained histological sections showing needle traces in the CdN. Red triangles indicate the estimated injection positions of the retrograde virus. Scale bar: 1 mm. CdN: caudate nucleus, IC: internal capsule, LV: lateral ventricle LPFC: lateral prefrontal cortex.

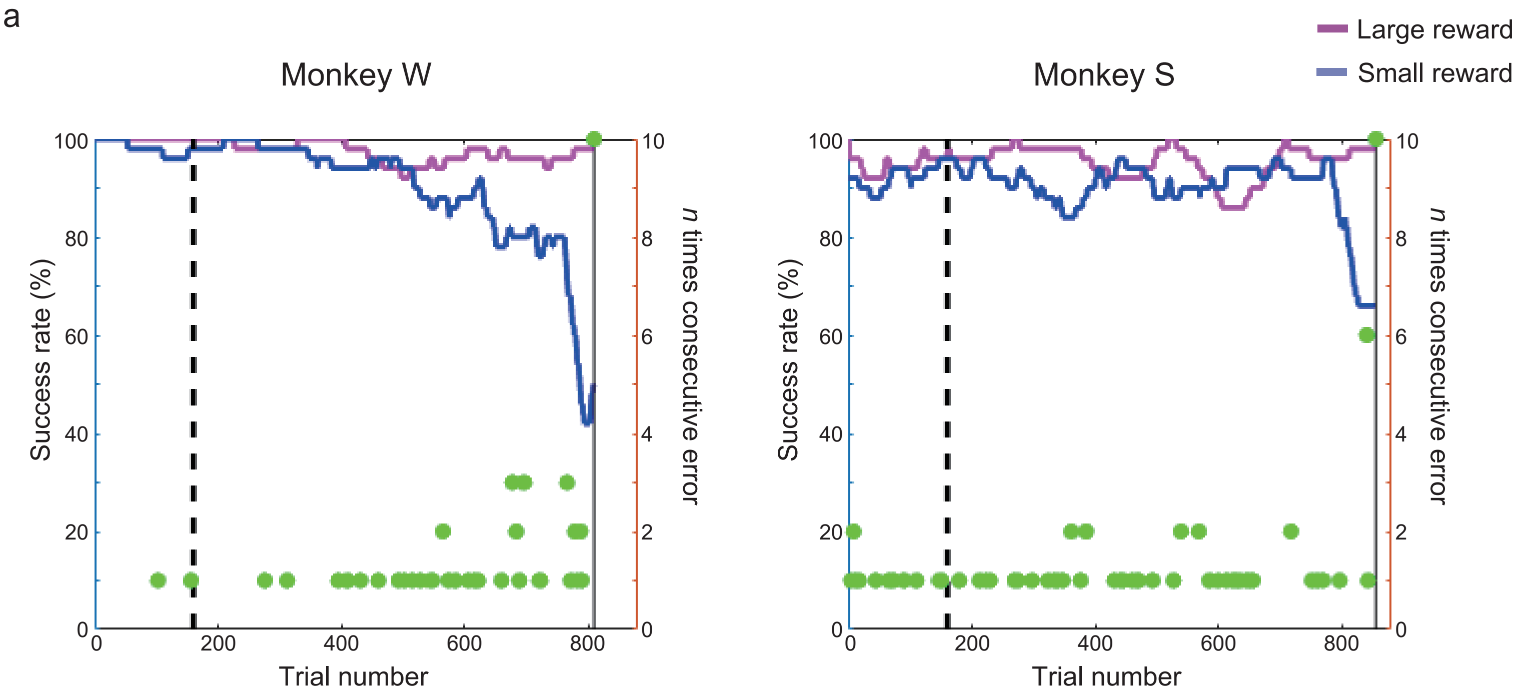

Supplementary figure 2 | Performance in example incomplete sessions in the CNO condition

(**a**) Behavioral performance in representative incomplete sessions for Monkey W (left) and Monkey S (right). Horizontal line indicates the trial number from the beginning of the session. The left-hand axis shows the success rate calculated over a 100-trial sliding window with a 1-trial step. The purple line indicates the success rate for large-reward trials and the blue line indicates that for small-reward trials. The right-hand axis shows n times consecutive errors indicated by green circles. The dotted vertical line refers to 160 trials from the start when CNO was administered. CNO: clozapine N-oxide.

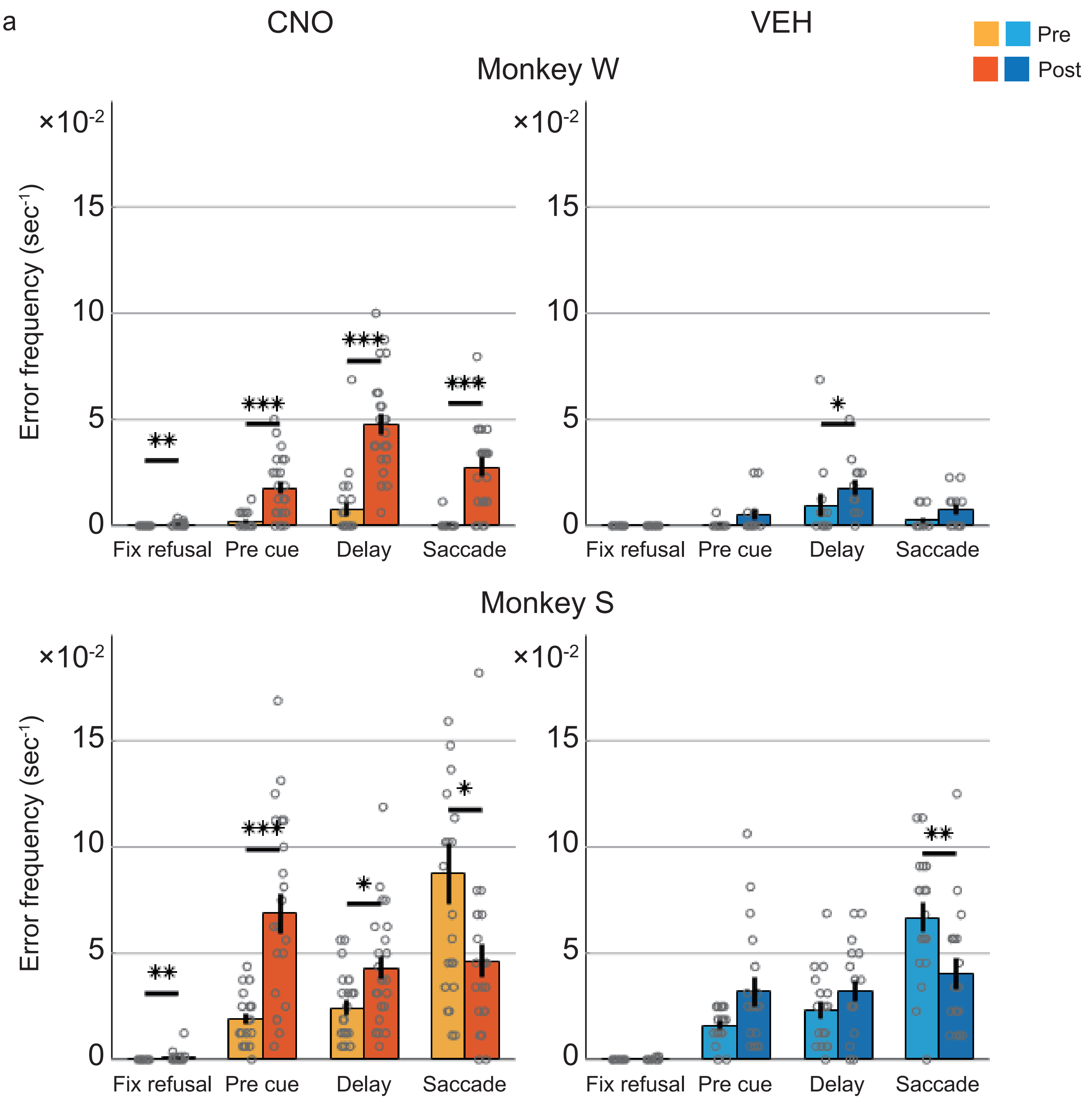

Supplementary figure 3 | Change in the frequency of each error type

(**a**) Frequency of four types of errors (Fix refusal: abort error before fixation, Pre cue: fixation break before the cue onset, Delay: fixation break during the delay period, Saccade: saccade error after the go signal), divided by condition, CNO (left) and VEH (right), for Monkey W (upper: CNO, Fix refusal: $p = 0.002$, Pre cue: $p < 0.001$, Delay: $p < 0.001$, Saccade: $p < 0.001$; VEH: Fix refusal: NaN, Pre cue: $p = 0.316$, Delay: $p = 0.017$, Saccade: $p = 0.089$, Wilcoxon rank-sum test) and for Monkey S (lower: CNO, Fix refusal: $p = 0.002$, Pre cue: $p < 0.001$, Delay: $p = 0.011$, Saccade: $p = 0.035$; VEH: Fix refusal: $p = 0.163$, Pre cue: $p = 0.130$, Delay: $p = 0.201$, Saccade: $p = 0.008$, Wilcoxon rank-sum test). Each dot indicates the error frequency per session for each condition. Pale colors: Pre. Deep colors: Post. Error bars: standard error of the mean. CNO: clozapine N-oxide, VEH: vehicle. NaN: Not a number.

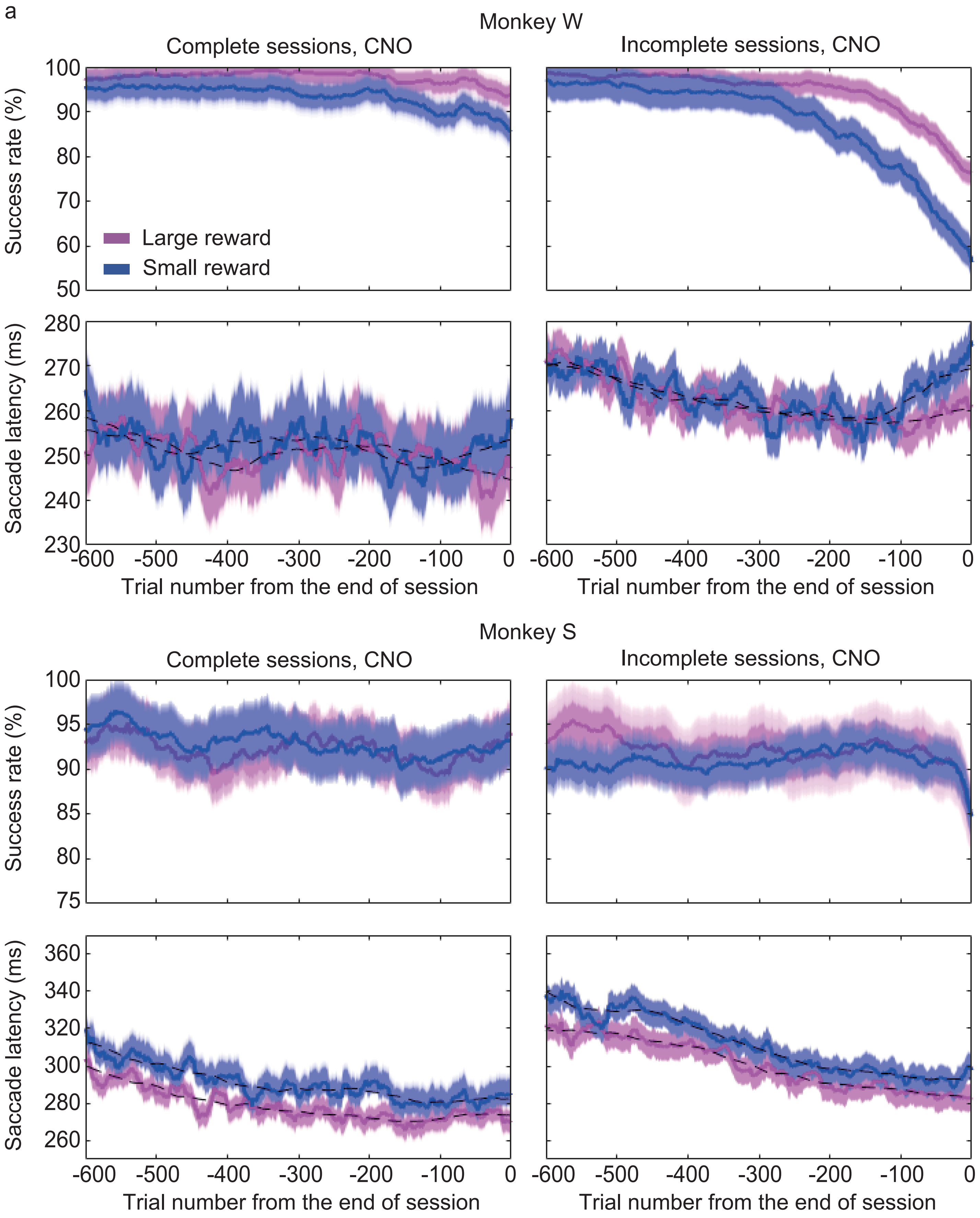

Supplementary figure 4 | Temporal changes in success rate and saccade latency on complete and incomplete sessions

(**a**) Success rate and saccade latency on complete and incomplete sessions in the CNO condition of Monkey W (Upper panels) and Monkey S (Lower panels). Horizontal line indicates the trial number from the end of

sessions. In each monkey, upper left: Success rate calculated over a 100-trial sliding window with a 1-trial step averaged over complete sessions in the CNO condition. The purple line indicates the success rate for large-reward trials and the blue line indicates that for small-reward trials. Upper right: Success rates for incomplete sessions. Lower left: The purple solid line indicates the moving average of saccade latency for large-reward trials in complete sessions with a 20-trial width and a 1-trial step and the blue solid line indicates that for small-reward trials. The dotted lines indicates corresponding moving averages with a 100-trial width. Lower right: Saccade latency for incomplete sessions. Shaded error bars: standard error of the mean. CNO: clozapine N-oxide.

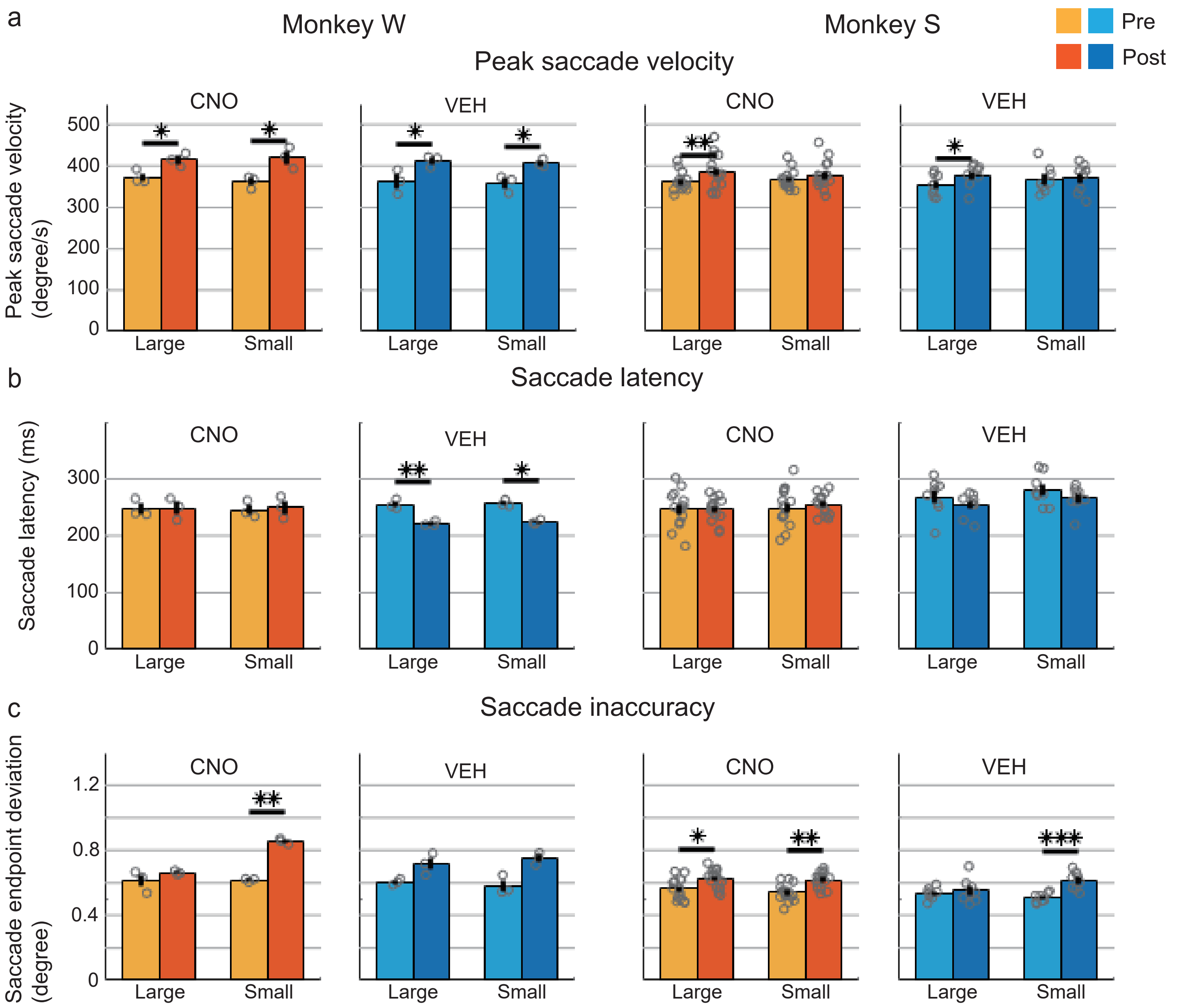

Supplementary figure 5 | Changes in saccade behavior before double virus transduction

(**a**) Peak saccade velocity after the go signal before double virus transduction. All features in these panels are the same as in Figure 3b. (**b**) Saccade latency. (**c**) Saccade inaccuracy.

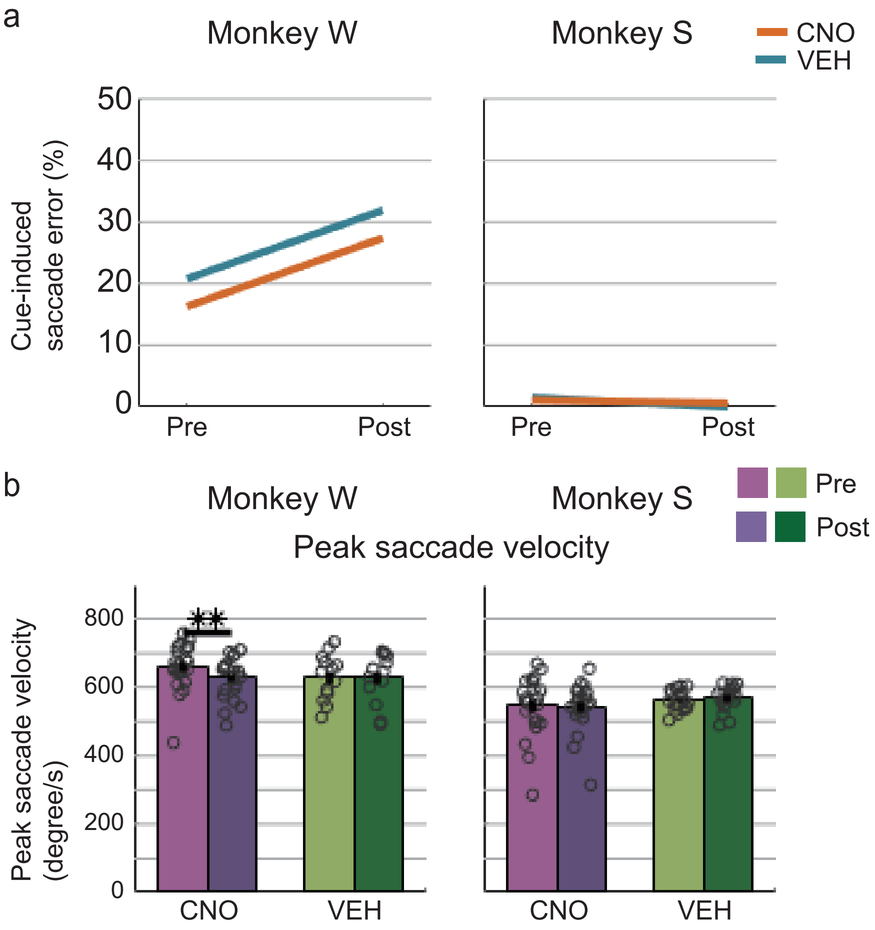

Supplementary figure 6 | No effects of CNO administration on response inhibition and saccades at the trial start

(**a**) Percentage of cue-induced saccade errors (saccadic fixation break toward the cue direction after cue onset) among all errors during the delay period for Monkey W (left) and Monkey S (right). Orange: CNO condition; Blue: VEH condition. (**b**) Peak saccade velocity at the trial start (after FP onset) for Monkey W (left) and Monkey S (right). Pale colors indicate the peak saccade velocity on Pre and deep colors indicate that on Post, separately calculated for CNO and VEH conditions. Each dot indicates the mean value per session for each condition. Error bars: standard error of the mean. CNO: clozapine N-oxide, VEH: vehicle, FP: fixation point.

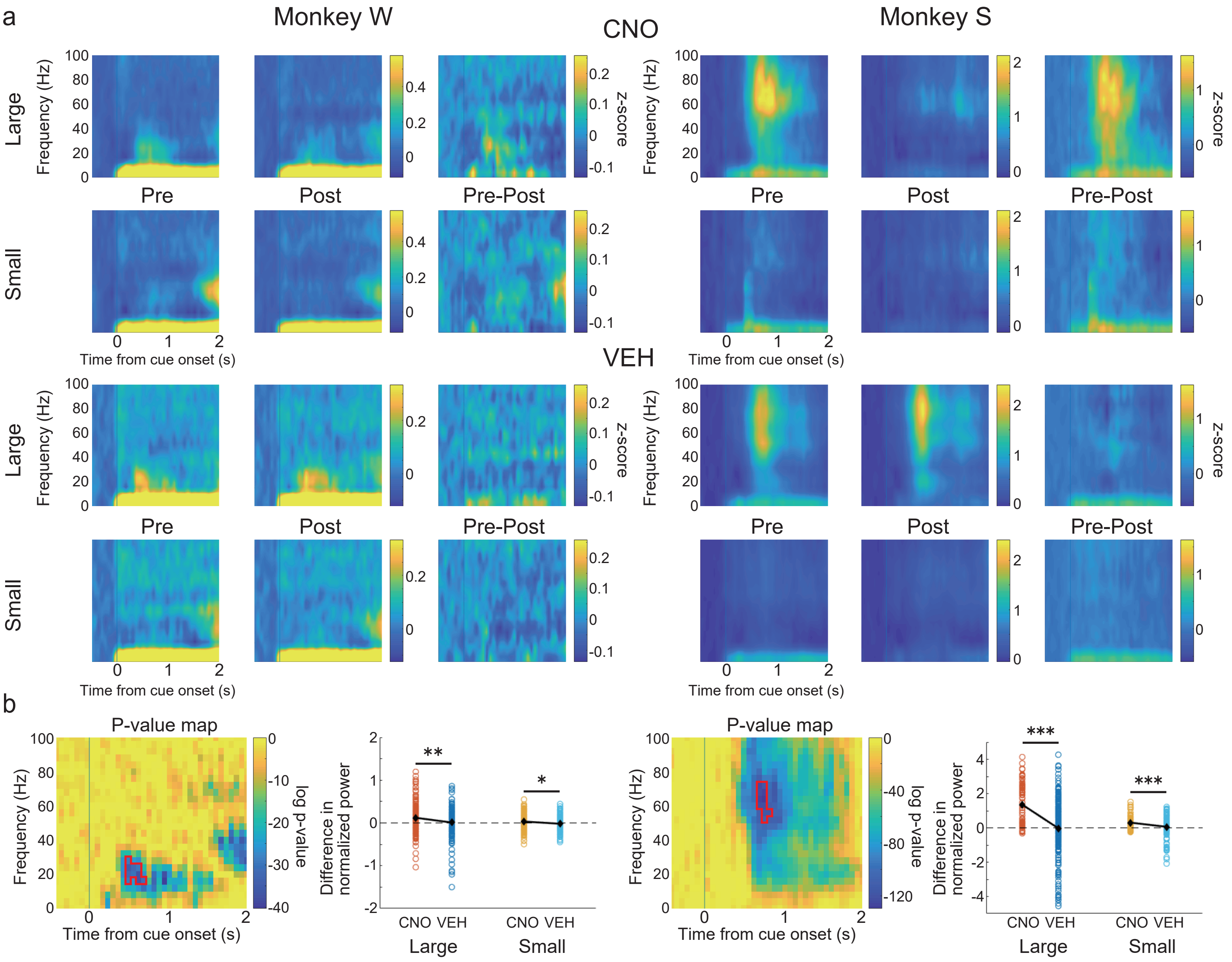

Supplementary figure 7 | Effects on spectrogram power in the PFC after cue onset (Contralateral)

(**a**) Normalized and averaged spectrograms of LFPs obtained from trials in which cue stimuli were presented to the contralateral visual field of the recording position in the LPFC for Monkey W (Left) and Monkey S (Right). All features in this panel are the same as in Figure 4a. (**b**) For each monkey: left: p-value map to extract ROIs; right: comparison of effects of CNO/VEH administration (Pre-Post, Monkey W, large: $t(319) = 2.34$, $p = 0.004$; small: $t(319) = 2.51$, $p = 0.013$; Monkey S, large: $t(306) = 6.84$, $p < 0.001$; small: $t(306) = 4.30$, $p < 0.001$, two-sample t-test). All features in this panel are the same as in Figure 4b. PFC: prefrontal cortex, CNO: clozapine N-oxide, VEH: vehicle, LFP: local field potential, LPFC: lateral prefrontal cortex, ROI: region of interest.

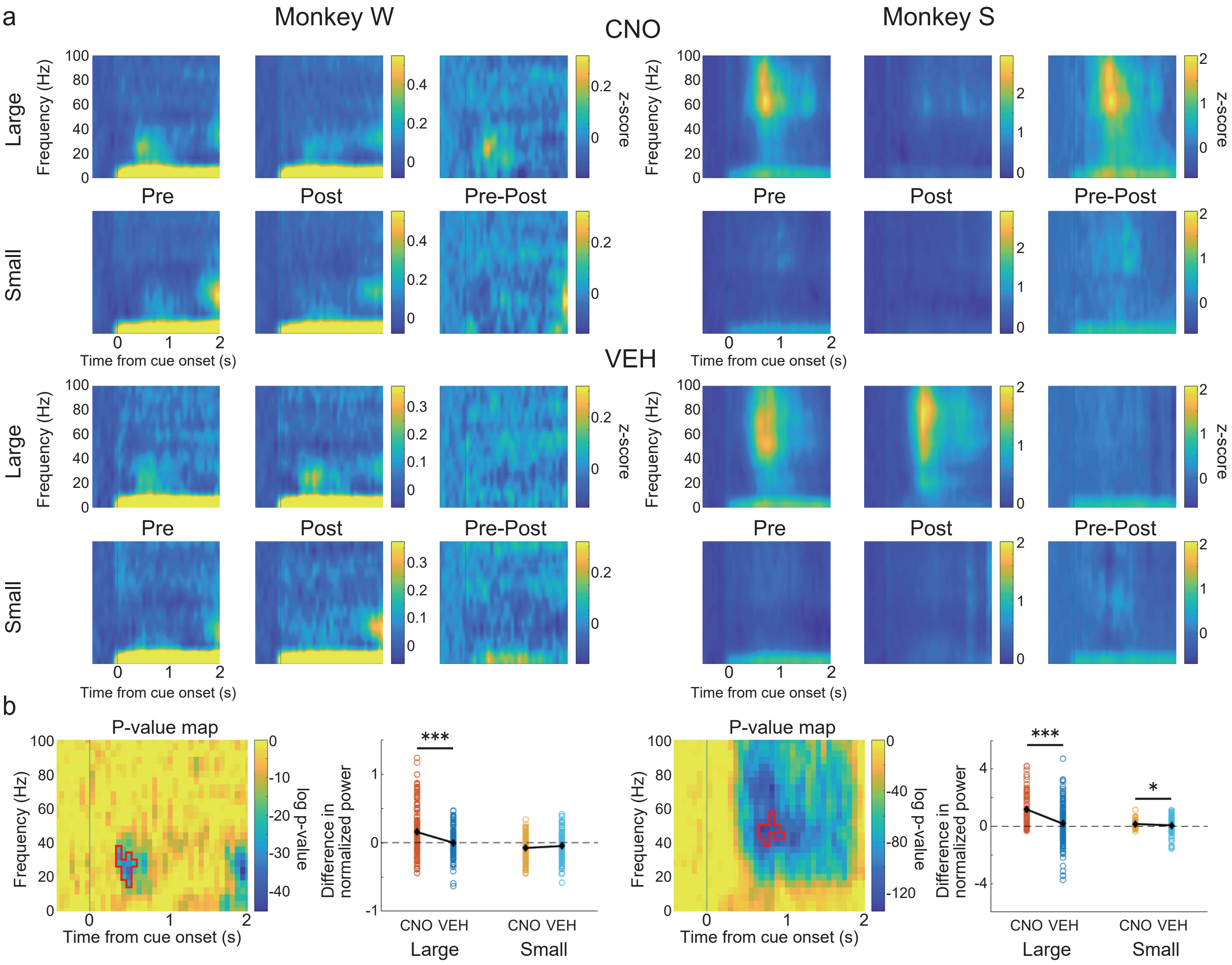

Supplementary figure 8 | Effects on spectrogram power in the PFC after cue onset (Ipsilateral)

(**a**) Normalized and averaged spectrograms of LFPs obtained from trials in which cue stimuli were presented to the ipsilateral visual field of the recording position in the LPFC for Monkey W (Left) and Monkey S (Right). All features in this panel are the same as in Figure 4a. (**b**) For each monkey: left: p-value map to extract ROIs; right: comparison of effects of CNO/VEH administration (Pre-Post: Monkey W, large: $t(319) = 5.04$, $p < 0.001$; small: $t(319) = 1.53$, $p = 0.128$; Monkey S, large: $t(306) = 6.75$, $p < 0.001$; small: $t(306) = 2.06$, $p = 0.041$, two-sample t-test). All features in this panel are the same as in Figure 4b. PFC: prefrontal cortex, CNO: clozapine N-oxide, VEH: vehicle, LFP: local field potential, LPFC: lateral prefrontal cortex, ROI: region of interest.

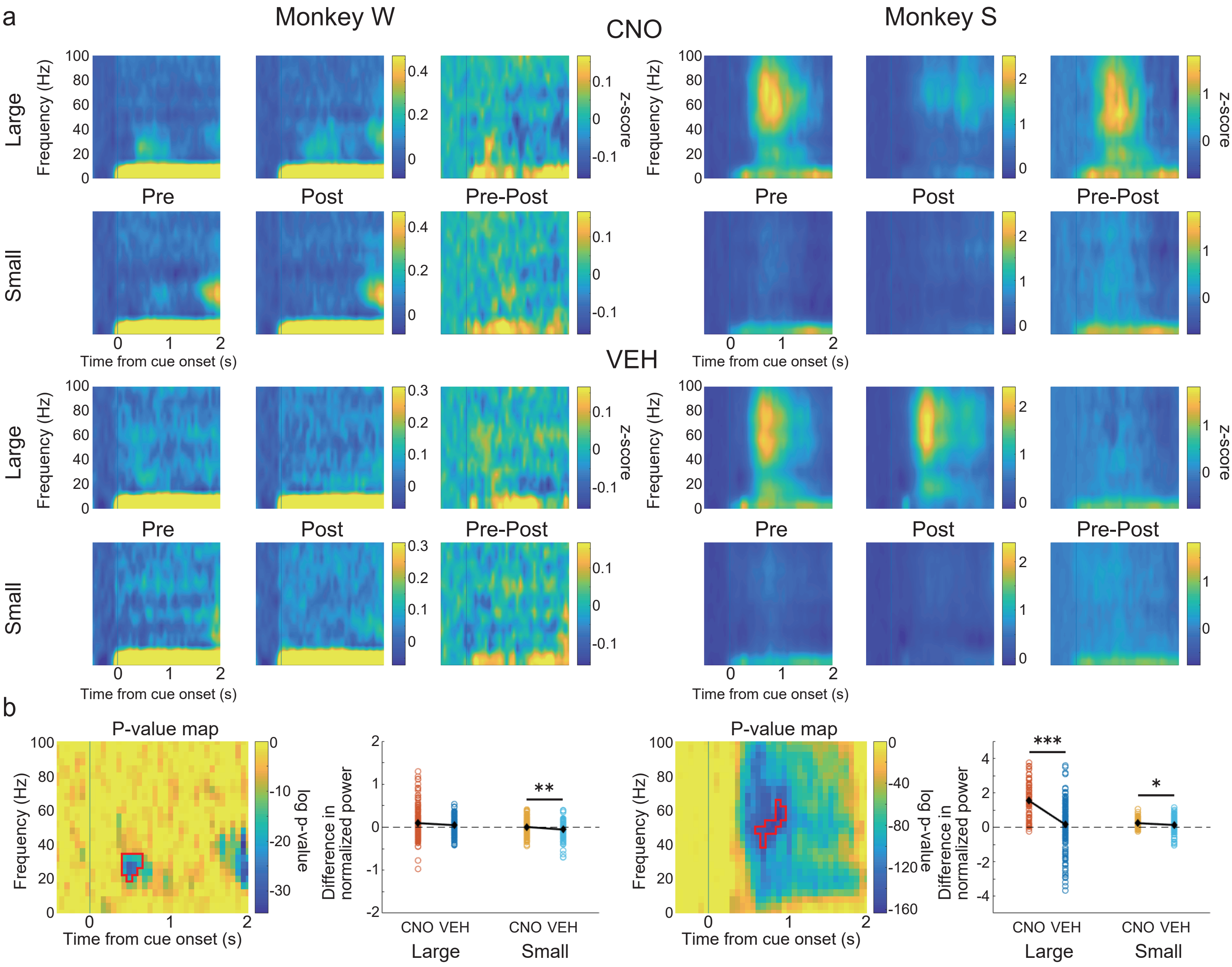

Supplementary figure 9 | Effects on spectrogram power in the CdN after cue onset (Contralateral)

(**a**) Normalized and averaged spectrograms of LFPs obtained from trials in which cue stimuli were presented to the contralateral visual field of the recording position in the CdN for Monkey W (Left) and Monkey S (Right). All features in this panel are the same as in Figure 4a. (**b**) For each monkey: left: p-value map to extract ROIs; right: comparison of effects of CNO/VEH administration (Pre-Post: Monkey W, large: $t(318) = 1.52$, $p = 0.130$; small: $t(318) = 3.01$, $p = 0.003$; Monkey S, large: $t(289) = 8.97$, $p < 0.001$; small: $t(289) = 2.52$, $p = 0.012$, two-sample t-test). All features in this panel are the same as in Figure 4b. CdN: caudate nucleus, CNO: clozapine N-oxide, VEH: vehicle, LFP: local field potential, ROI: region of interest.

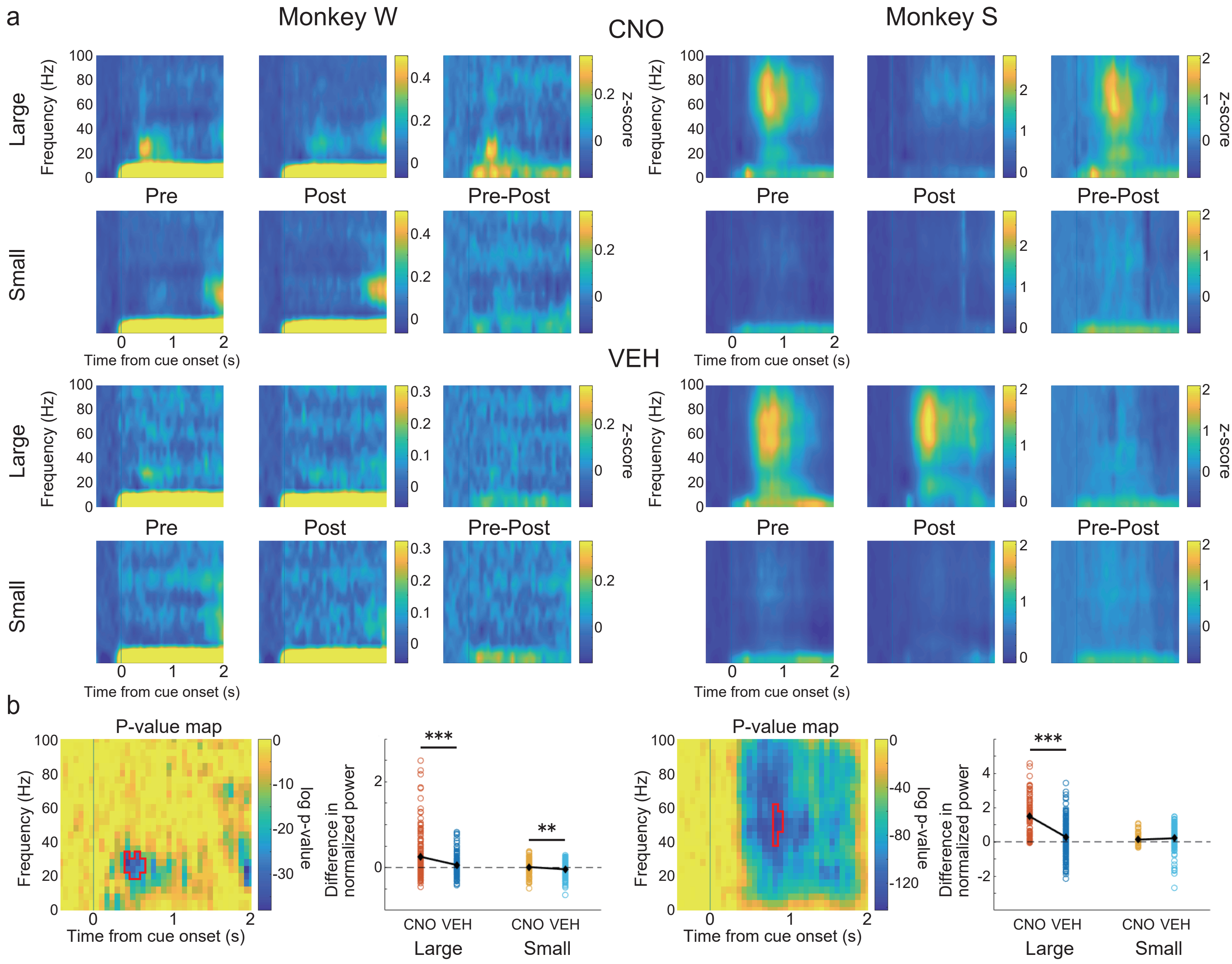

Supplementary figure 10 | Effects on spectrogram power in the CdN after the cue onset (Ipsilateral)

(**a**) Normalized and averaged spectrograms of LFPs obtained from trials in which cue stimuli were presented to the ipsilateral visual field of the recording position in the CdN for Monkey W (Left) and Monkey S (Right). All features in this panel are the same as in Figure 4a. (**b**) For each monkey: left: p-value map to extract ROIs; right: comparison of effects of CNO/VEH administration (Pre-Post: Monkey W, large: $t(318) = 4.30$, $p < 0.001$; small: $t(318) = 2.85$, $p = 0.005$; Monkey S, large: $t(289) = 9.10$, $p < 0.001$; small: $t(289) = 1.20$, $p = 0.232$, two-sample t-test). All features in this panel are the same as in Figure 4b. CdN: caudate nucleus, CNO: clozapine N-oxide, VEH: vehicle, LFP: local field potential, ROI: region of interest.

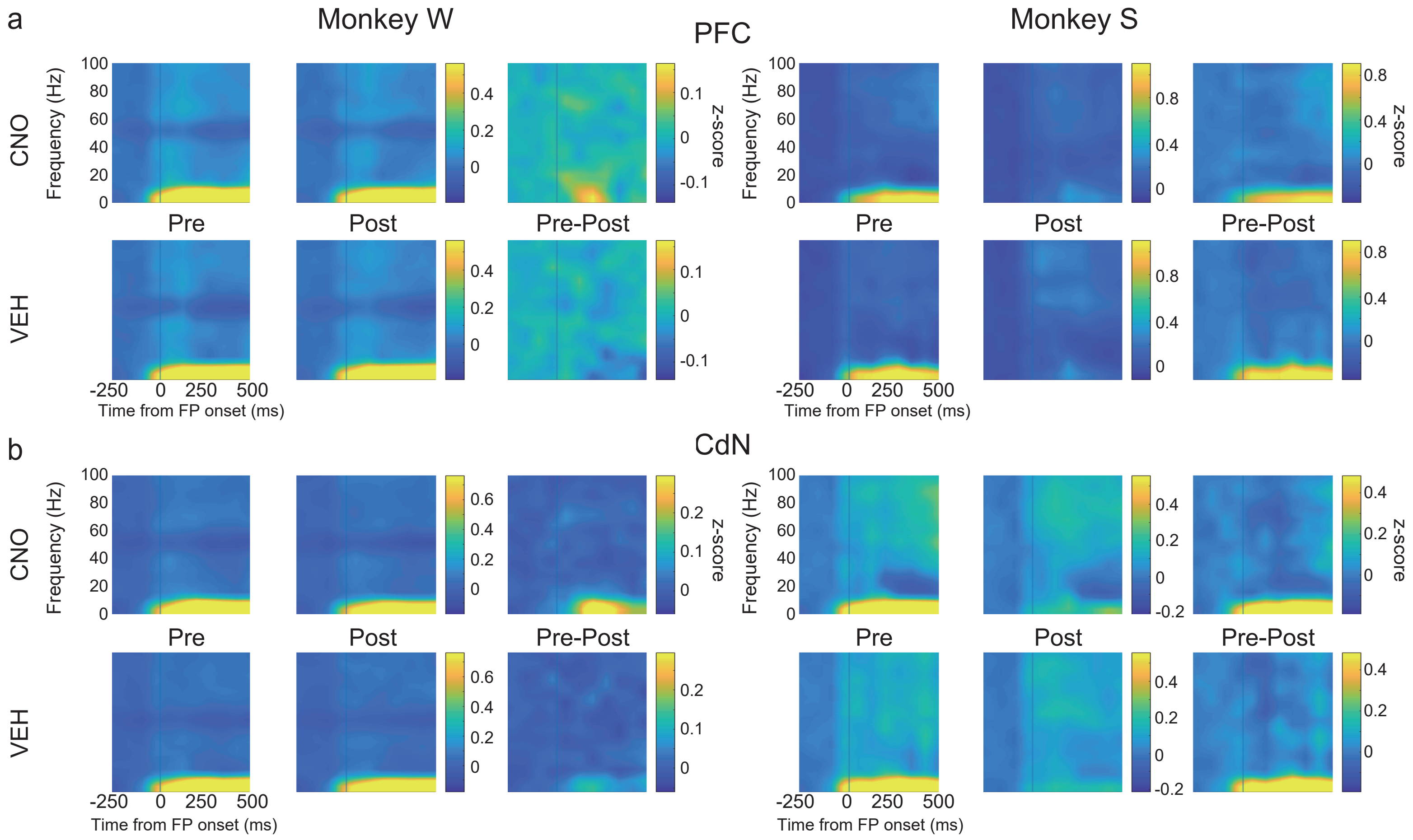

Supplementary figure 11 | Spectrogram power after FP onset

(**a**) Normalized and averaged spectrograms of LFPs from electrodes in the LPFC for Monkey W (Left) and Monkey S (Right), divided into the CNO (upper) and VEH (lower) conditions. Time 0 refers to the FP onset. All other features in these panels are the same as in Figure 4a. (**b**) Normalized and averaged spectrograms of LFPs from electrodes in the CdN. LPFC: lateral prefrontal cortex, CdN: caudate nucleus, CNO: clozapine N-oxide, VEH: vehicle.

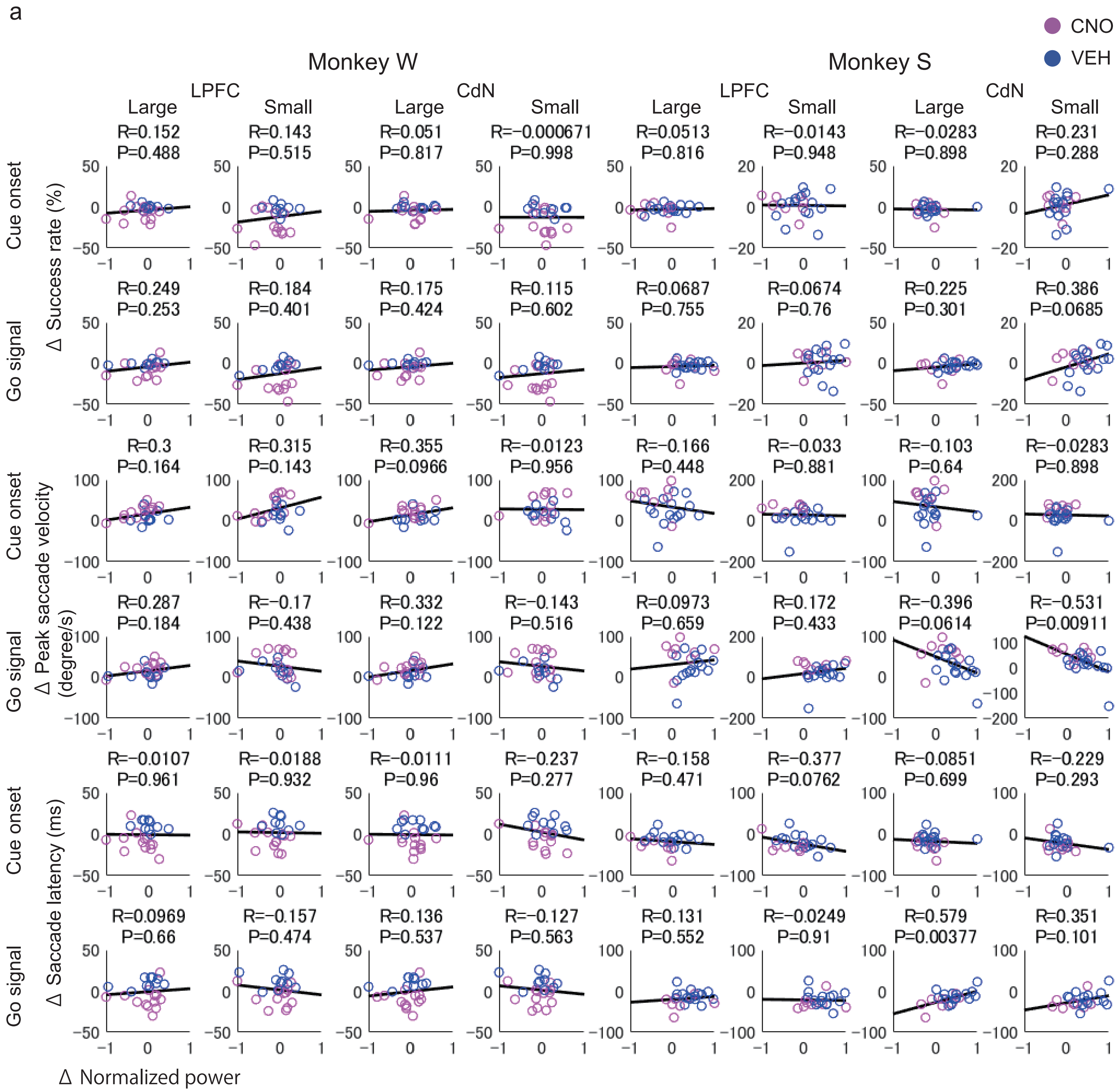

Supplementary figure 12 | Correlation between LFP and behavioral changes.

(**a**) Scatterplots of the difference in LFP mean power within the extracted ROIs (Post-Pre) and that in behavioral parameters (Post-Pre). The columns are Pre/Post differences of LFP: the left four columns are from Monkey W and the right four columns are from Monkey S. The left two columns of each monkey are large and small reward trials recorded in LPFC and the right two columns are those recorded in CdN. The rows are the Pre/Post differences in behavioral parameters: the top two rows are success rate, the middle two are the peak saccade velocity, and the bottom two are saccade latency, respectively. Within them, the top row is after the cue onset and the bottom is after the go signal. The purple circles represent CNO sessions, and the blue circles represent VEH sessions. The solid black line is the regression line. R above each scatter plot represents the

Pearson correlation coefficient and P represents the p-value. LPFC: lateral prefrontal cortex, CdN: caudate nucleus, CNO: clozapine N-oxide, VEH: vehicle.
