# Supplementary material for: Chemogenetic inactivation reveals the inhibitory control function of the prefronto-striatal pathway in the macaque brain
Source: Commun Biol. 2021 Sep 16;4:1088. doi: 10.1038/s42003-021-02623-y (PMC8446038; doi:10.1038/s42003-021-02623-y)
Supplement: Supplementary file 3 — Description of Additional Supplementary Files [file 42003_2021_2623_MOESM3_ESM.pdf]

Description of Additional Supplementary Files

File name: Supplementary Data 1

Description: An excel file containing the data of main figures (Figures 1-8).

File name: Supplementary Data 2

Description: An excel file containing the data of supplementary information (Supplementary figures 1-12).
